# Comparison between Ultrasound-Guided and Palpatory Localization of the Dorsal Joint Space of the Shoulder Joint

**DOI:** 10.3390/diagnostics14060650

**Published:** 2024-03-20

**Authors:** Stephan Stein, Andreas Weimer, Svenja Berthold, Johannes Matthias Weimer, Arnold J. Suda, Christopher Tuffs, Gerhard Schmidmaier, Christian T. Schamberger

**Affiliations:** 1 Clinic for Trauma and Reconstructive Surgery, University Clinic Heidelberg, 69118 Heidelberg, Germany; andreas.weimer@kkh-bergstrasse.de (A.W.);; 2 Department for Orthopaedics and Trauma Surgery, University Medical Centre Mannheim, 68167 Mannheim, Germany; 3 Rudolf Frey Learning Clinic, University Medical Centre of the Johannes Gutenberg University Mainz, 55131 Mainz, Germany; 4 Department of Orthopaedics and Trauma Surgery, AUVA Trauma Center Salzburg, 5010 Salzburg, Austria; 5 Department of General, Visceral, Thoracic and Transplantation Surgery, University of Giessen, 35390 Giessen, Germany

**Keywords:** ultrasound, shoulder puncture, shoulder joint puncture, musculoskeletal ultrasound, arthroscopy, ultrasound-guided procedure, video-based learning, physical examination

## Abstract

Aim of the study: Arthroscopy ranks among the frequently performed interventions in orthopedics. The aim of this study was to compare the palpation technique with the ultrasound technique for locating the dorsal glenohumeral joint space (JS) in shoulder joint punctures. Material and Methods: Participants inexperienced in ultrasound examinations were included. Palpatory and ultrasound finding of the joint space by the participants was performed according to current recommendations and was initially demonstrated by an instructional video. The ideal point (IP) was marked under ultrasound visualization by an experienced ultrasound examinator and shoulder–elbow surgeon. Furthermore, a corridor for a safe puncture was defined. The palpatorily determined point (pdP) was marked by the participants and evaluated by means of a coordinate system. The evaluation of the sonographically determined point (sdP) was performed similarly to that of the palpatory procedure. Results: Fifty-four participants were included in the study, and the mean length of work experience was 6.3 years. On average, participants had performed 16.5 punctures of the shoulder joint and 6.8 arthroscopies of the shoulder joint. The mean experience in performing sonographic examinations of the shoulder was 27.6 examinations. A total of 100 shoulder joints were examined (54 left, 46 right shoulders). The mean deviation from the ideal point (IP) for the palpatory approach was 17.1 mm with a maximum deviation of 59.5 mm; for the sonographic technique, the mean deviation was 10.3 mm (max. 30.2 mm). Overall, 22% of pdPs were within the defined corridor, while 42% of sdPs were within the target corridor. The average difference between palpatory and sonographic approaches was 9.0 mm in favor of the sonographic technique (max. 46.5 mm). A significantly greater deviation (*p* < 0.001) from the IP was observed with the palpatory approach than with the sonographic approach. Conclusion: Based on the results, the authors recommend ultrasound imaging of the shoulder joint as well as ultrasound-assisted punctures, especially for inexperienced users. Furthermore, training in ultrasound-assisted interventions should be implemented in future training curricula.

## 1. Introduction

Puncture and injection of joints constitute an established routine practice, especially in the fields of orthopedics, trauma surgery, rheumatology, and radiology. These procedures serve for both the diagnosis and therapy of acute and chronic joint diseases [1,2,3]. A recent study shows that the sonographic approach for arthrocentesis is superior to the conventional palpatory method in medium-sized joints such as the elbow, wrist, and ankle [4].

However, due to the different morphology of the acromion process and possibly extensive soft tissue around the glenohumeral joint, joint puncture can be a challenge [5]. Typically, shoulder puncture is performed dorsally, at the same location as the dorsal standard portal for initiating shoulder arthroscopy. The correct positioning of the portal is crucial for a successful arthroscopic operation. This standardized approach allows the surgeon to inspect all relevant joint structures and perform interventions under optimal conditions [2,6,7,8]. Even minor deviations from the precise position can lead to suboptimal results [9]. Incorrect positioning of the puncture needle can not only cause nerve and vessel damage but also result in inadequate aspiration of material, also known as “punctio sicca” [2]. This phenomenon can occur during infiltration therapy and lead to insufficient therapeutic effects of the injected medication. Such misplacements can also occur during planned injections of contrast agents for imaging procedures such as MRI (arthro-MRI) and potentially cause local side effects. Complications during arthrocentesis such as vascular and nerve injuries in general are rare; however, the most feared complication is an infection of the joint [10,11].

The palpatory localization of the joint gap is usually achieved by identifying the transition from the scapular spine to the acromion. This localization can be a challenging task due to anatomical variations [12,13,14]. Due to the different acromion shapes in the acromion process (triangular, quadrangular, and tubular) palpatory detection of the posteriolateral corner as a starting point for orientation for locating the dorsal joint space can cause difficulties, and this can lead to incorrect punctures [5].

Starting from this dorsolateral transition, the joint gap is located 2 cm caudal and 1 cm medial to this position. Lack of orientation in this localization can lead to incorrect placement of instruments, with potentially serious complications, including damage to peri- or intra-articular structures and the aforementioned punctio sicca [2,15].

Ultrasound-guided detection of the shoulder joint space is an alternative technique. The main trend in the comparative selection of these two techniques is that, in everyday clinical practice, the palpatory technique is generally used to locate the joint space in the shoulder from the dorsal side, both for arthroscopy and for joint puncture. An alternative to the palpatory technique is image-guided detection of the joint space, which is usually only carried out using computer tomography or X-ray and has the disadvantage of being a time-consuming, cost-intensive, and, above all, radiation-intensive examination.

In their study, Sibbit et al. were able to show that ultrasound-guided aspiration and application of corticosteroids to the knee joint are associated with significantly less pain and increased aspiration volume of synovial fluid compared to the conventional method using anatomical landmarks [16]. In contrast, Wiler et al. showed in their prospective, randomized study that there was no significant difference in the overall success of joint fluid aspiration compared to the standard technique (landmark), but the ultrasound-guided puncture was not more painful for the patient, did not require any additional time, and led to greater fluid aspiration by inexperienced physicians and greater confidence in the procedure on the part of the inexperienced physician [17].

The ultrasound-guided puncture or injection involves three different approaches according to the German guidelines of the AWMF [18].

In the first method, the joint to be punctured is initially visualized sonographically, and the corresponding location, i.e., the joint space, is marked during the sonographic examination. Subsequently, the ultrasound transducer is removed, and the puncture is performed according to standard hygiene protocols, including disinfection and sterile covering. This method involves a puncture after the sonography has been conducted.

Another method involves the use of specialized puncture ultrasound transducers. Various models are available to the examiner. Some transducers have an opening through which the puncture needle is guided and then visualized in the ultrasound image. Additionally, depending on the ultrasound device model, clips are available that can be attached to the ultrasound transducer. The puncture needle is guided through the attached clip, ensuring its position under the transducer. Sterile covers for the respective devices are necessary for both methods. Sterile-packaged ultrasound gels are used as coupling media. Alternatively, disinfectants for the skin can be used. This approach involves a puncture during sonography.

The last method involves using a standard ultrasound transducer. In this case, the puncture needle must pierce the skin at a safe distance from the transducer while maintaining a specific angle for visualization of the needle under the transducer. This method typically requires both steady transducer handling and a secure puncture technique. To maintain hygiene, sterile covers, similar to those used in intraoperative sonographic procedures, can be used as an alternative. This method also involves a puncture during the sonographic examination.

In summary, ultrasound examination demonstrates great potential as a valuable tool for successful and sufficient arthrocentesis of the glenohumeral joint due to its cost effectiveness, avoidance of ionizing radiation, and capability for dynamic imaging [19,20]. However, further research and validation studies are needed to establish ultrasound-assisted puncture of the shoulder as a safe and practicable method, especially for inexperienced physicians.

Considering these challenges, the primary objective of this study was to compare the palpatory and ultrasound-guided methods in identifying the dorsal glenohumeral joint gap for joint punctures and in placing a standardized dorsal portal. Additionally, it should be analyzed whether less experienced doctors could achieve better results through the ultrasound-guided method compared to the purely tactile approach.

## 2. Materials and Methods

### 2.1. Study Design

This prospective clinical superiority study was developed at the Center for Orthopedic and Trauma Surgery, University Hospital Mannheim. The ethics vote was obtained from the Ethics Committee of the Medical Faculty Mannheim, University Heidelberg (application number: 2020-615N).

The primary endpoint of the present study was to investigate whether ultrasound-guided puncture technique is superior compared to the palpation approach in the shoulder joint. Clinical significance refers to whether the ultrasound-guided puncture technique provides better outcomes or benefits for patients compared to the palpation approach. This encompasses factors such as improved accuracy of needle placement and decreased risk of complications, which correlate with enhanced therapeutic efficacy.

Statistical significance, on the other hand, was determined through rigorous statistical analysis of the collected data.

The secondary endpoint of this study aimed to investigate whether less experienced physicians performing sonographically guided shoulder joint puncture show a significantly improved accuracy compared to when performing the conventional palpation technique.

### 2.2. Participant Recruitment and Inclusion and Exclusion Criteria

The following prospective study was conducted with voluntary participants of a DEGUM (German Society of Ultrasound in Medicine)-, ÖGUM (Austrian Society of Ultrasound in Medicine)-, and SGUM (Swiss Society of Ultrasound in Medicine)-certified ultrasound course for the musculoskeletal system. Inclusion criteria required participants to be approbated in Germany, Austria, or Switzerland and to have completed or be currently completing their residency for orthopedics and trauma surgery, radiology, rheumatology, general medicine, rehabilitation, and physical medicine. Furthermore, only participants with little to no experience in arthroscopy or puncture of the glenohumeral joint (*n* < 100) were included, referring to the training guidelines for Orthopedic and Trauma Surgery of the Federal Medical Association of Germany [21].

Moreover, the statistical analysis included only participants who conducted both the sonographically guided and palpation methods on at least one shoulder joint during the examination. Those who utilized only the sonographic or palpation method exclusively were excluded from the study.

Participants with incomplete datasets regarding professional experience, independently performed arthrocentesis of the shoulder joint, or independently conducted ultrasound examinations, etc., were also not included in the analysis.

### 2.3. Study Procedure, Materials, and Evaluation

**Figure 1:**
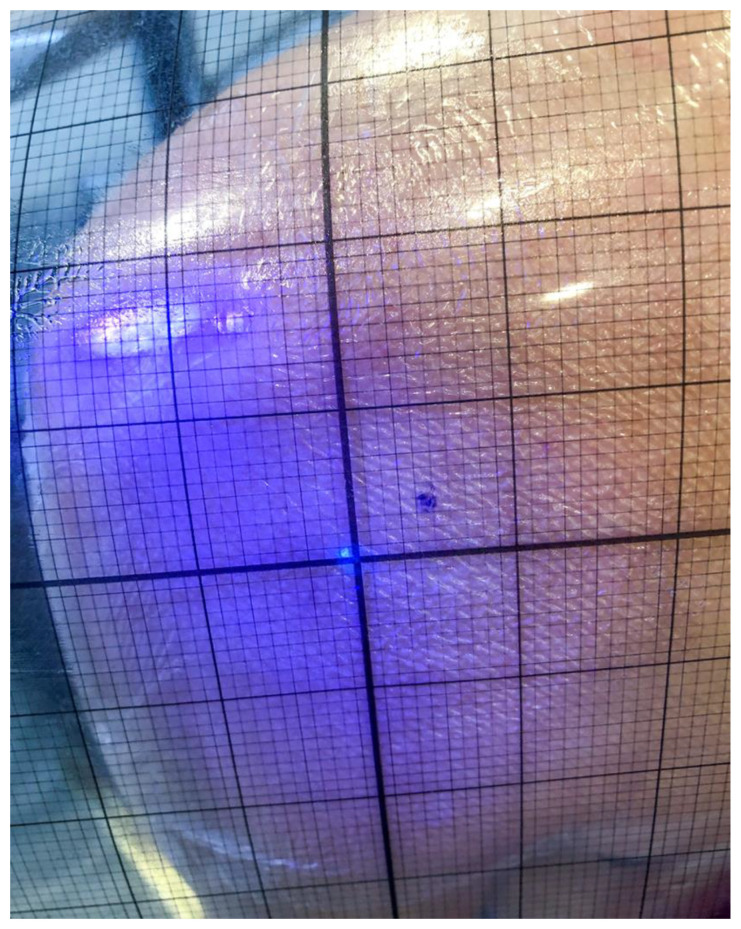
Determination of the deviation from the reference/ideal point on the left shoulder with a UV pen and covered with a transparent foil with millimeter markings.

First, the dorsal joint gap was defined by a DEGUM- and DVSE (German Society for Shoulder and Elbow)-certified shoulder–elbow surgeon experienced in both arthroscopy and sonography by visualization using sonography, conducted multiple times to ensure the correct position. This point was defined as the ideal point (IP). It was marked using a UV pen and covered with transparent foil. Four quadrants were defined and numbered using a coordinate system starting from the IP, while the craniolateral quadrant was defined as 1, the caudolateral quadrant as 2, the caudomedial quadrant as 3, and the craniomedial quadrant as 4. Furthermore, a rectangular corridor above the IP, in which puncture of the joint could be performed, as well as safe insertion of a trocar for arthroscopy, was defined, located between 1 cm above and 2 cm below, as well as 0.5 cm to the left and right, of the IP (Figure 1).

#### 2.3.1. Palpatory Localization of the Dorsal Joint Space

**Figure 2:**
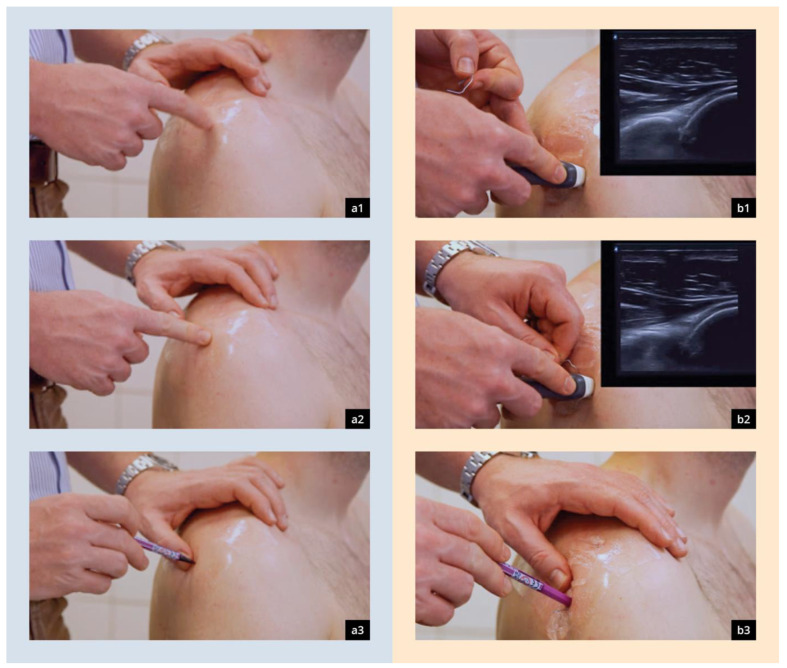
Presentation of the PalpMethod (**a1**–**a3**) and SonoMethod (b + d) for determining the optimal access points to the joint spaces [24]. (**a1**) Identification of the posteriolateral corner of the acromion, (**a2**) palpation of the dorsal “Soft Spot”, (**a3**) highlighting of the spot with a marker pen, (**b1**) obtaining the dorsal transverse view of the shoulder, (**b2**) placement of the paperclip acoustic shadow above the joint, (**b3**) highlighting of the spot with a marker pen.

After consenting to the study, participants were introduced to the palpatory method by an instructional video. Palpatory localization of the joint gap was conducted as recommended by the current guidelines—starting from the angulus acromii 2 cm distally and 1 cm medially [22]. The point determined by palpation (pdp) was marked on the foil and, in the following, analyzed using a coordinate system. The previously marked correct position of the joint gap (IP) was also visualized using a UV light. Participants were not informed about the point they determined by palpation (Figure 2b).

#### 2.3.2. Ultrasound-Guided Localization of the Dorsal Joint Space

The method of puncture following sonography was employed for the execution of the present study. A meticulous selection of this specific technique was made as it represents a practical approach in everyday settings that requires no specialized equipment. Ultrasound-guided marking was conducted using the dorsal transversal plane as recommended by DEGUM guidelines [20,23] with the help of a paperclip as described by Konermann et al. [24]. The described method was previously demonstrated to the participants by an instructional video. Ultrasound was conducted using a plane transducer with variable frequencies ranging from 7 to 15 MHz. Initially, the glenohumeral gap joint, as well as the optimal cranio–caudal alignment, was determined using the correct standard plane. Subsequently, a bent-up paperclip was positioned at the optimal medio–lateral alignment underneath the linear transducer and fixated. The linear transducer was removed and the joint gap marked again (point estimated by ultrasound = sdp). Analysis of the sdp was conducted similarly to the previously described palpatory procedure (Figure 2b).

### 2.4. Cut-Off for Experienced Participants

The cut-off for experienced participants was defined as *n* < 20 punctures and arthroscopies of the shoulder. This was based on the study by O’Neill et al., which postulated minimal competence in shoulder arthroscopy from 23 + 24.7 (range, 3–150) procedures [25]. Since participants only estimated their number of arthroscopies performed, the cut-off value was set at 20.

### 2.5. Statistical Analysis

The data obtained were entered and processed manually using MS Excel. Statistical analysis was performed using the statistical software SPSS software, version 27 (IBM SPSS Statistics for iOS, Armonk, North Castle, NY, USA).

Binary and categorical baseline parameters are expressed as absolute numbers and percentages. Continuous data are expressed as median and interquartile range (IQR), or as mean and standard deviation (SD). Data distribution was tested using the Shapiro–Wilk test. The mean values of the diagonal of the palpatory method and the sonographic method were compared. Statistical parameters were calculated for both methods for the left and right shoulders, and analyses of variance were performed. As the variables were not normally distributed, categorical parameters were compared using Fisher’s exact test and continuous parameters using the Mann–Whitney test. Pearson’s correlation coefficient was determined, and a Maloney and Rastogi test was performed. Precision differences were represented by a Bland–Altman plot. The quadrants were compared descriptively and visualized graphically by boxplots. A chi-squared test checked the error variance of the quadrants. *p* values < 0.05 were considered statistically significant.

## 3. Results

### 3.1. Demography

A total of 72 individuals participated in the present study, during which 144 measurements were conducted. A total of 54 participants were eligible and included in the study. After careful application of the specified inclusion and exclusion criteria, 100 measurements were ultimately included in the final analysis.

The mean length of job experience was 6.3 years (±4.2 years). On average, participants had conducted 16.48 (±28.7) punctures and 6.76 (±18.5) arthroscopies of the glenohumeral joint. The mean experience (number of examinations) of independent ultrasound-guided examinations of the glenohumeral joint was 27.6 (±75.9).

In total, this identified participants with prior experience in arthroscopy (*N* = 11), shoulder puncture (*N* = 21), and shoulder sonography (*N* = 19). A total of 100 shoulder joints were examined, while examinations were evenly distributed with 54 left and 46 right shoulders. In line with the inclusion criteria, only one shoulder joint was sonographically and palpatorily examined by eight participants.

### 3.2. Palpatory Localization (PalpMethod)

**Figure 3:**
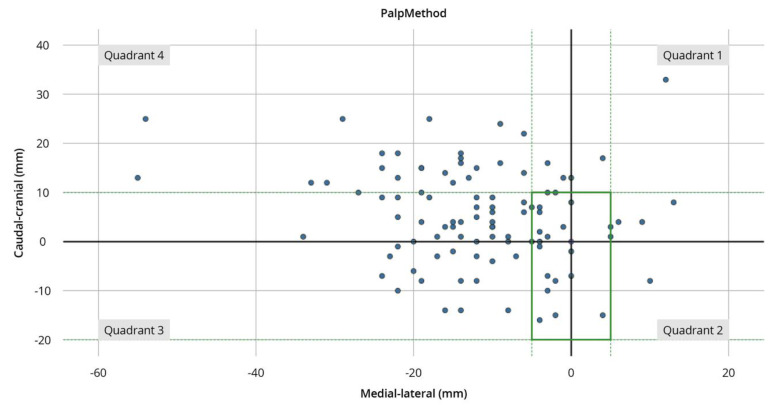
Scatter plot of the palpatory localization: The IP is visualized as the red dot in the center. The green dotted lines show cranio–caudal and medio–lateral borders of the defined corridor for secure puncture of the shoulder joint. The green solid line represents the corridor itself. The blue dots show the marks for a potential puncture of the participants. The *y*-axis shows the cranio–caudal orientation. The *x*-axis shows the medio–lateral orientation.

**Figure 4:**
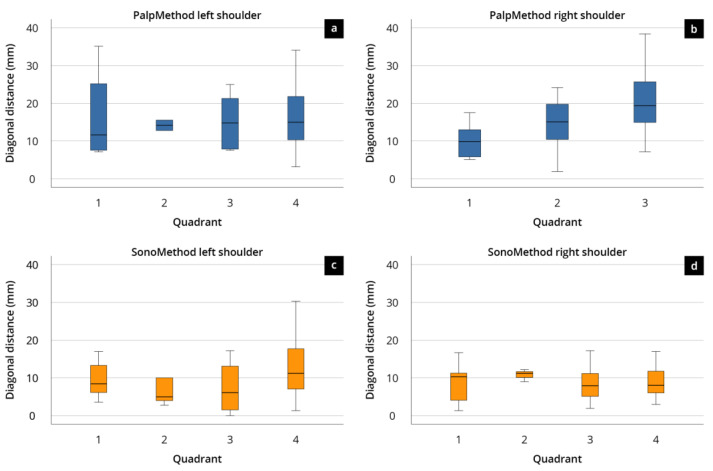
Boxplot presentation of the quadrant deviations (quadrants 1–4) of the performed palpatory and sonographic markers; craniolateral quadrant was defined as 1, the caudolateral quadrant as 2, the caudomedial quadrant as 3, and the craniomedial quadrant as 4.

The mean deviation from the IP was 17.12 mm (±9.95 mm) with a maximum deviation of 59.51 mm after palpation, while ultrasound-guided estimation deviated on average by 10.29 mm (±6.09 mm) with a maximum deviation of 30.23 mm. Regarding the defined quadrants, most points estimated by palpation (pdp) were located in the fourth quadrant (64%) and the fewest in quadrant 2 (2%) (Figure 3 and Figure 4).

### 3.3. Ultrasound-Guided Localization (SonoMethod)

**Figure 5:**
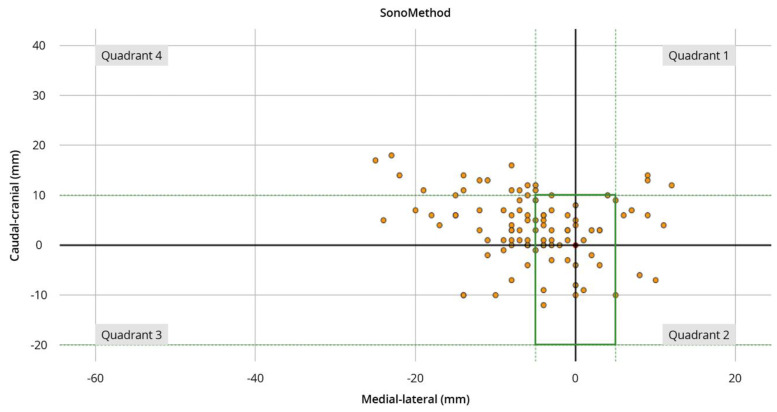
Scatter plot of the sonography-assisted localization of the IP (red dot). The green dotted lines show cranio–caudal and medio–lateral borders of the defined corridor for secure puncture of the shoulder joint. The green solid line represents the corridor itself. The orange dots show the marks for a potential puncture of the participants. The *y*-axis shows the cranio–caudal orientation. The *x*-axis shows the medio–lateral orientation.

Similarly, in the ultrasound group, most sdps were identified in quadrant 4 (61%) and the fewest in quadrant 2 (8%). In total, 22% of points defined by palpation were in the defined corridor, whereas 42% of points estimated by ultrasound-guided examination were located correctly. Inspection of the quadrants within the defined corridor showed that sdps were located with 3% in quadrant 1, 2% in quadrant 2, 8% in quadrant 3, and 10% in quadrant 4. Sdps were located with 8% in the first, 6% in the second, 9% in the third, and 19% in the fourth quadrant (Figure 4 and Figure 5).

### 3.4. Comparative Statistical Analysis

**Figure 6:**
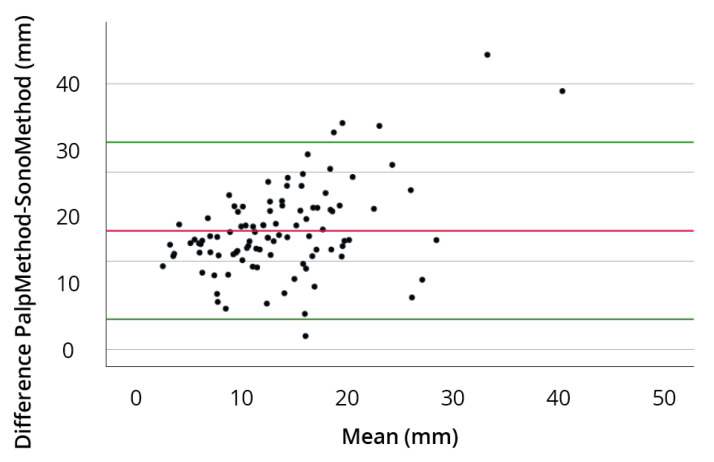
Bland–Altman plot: The *x*-axis shows the mean values of the measurements from both methods. The *y*-axis reflects the differences between the measurements of both methods. Red line shows the mean of the difference, green lines show the mean of the difference + 1.96 × SD of the difference.

The mean difference between the palpatory and ultrasound-guided approaches was 9.045 (±8.268) in favor of the ultrasound-guided technique, with a maximum deviation of 46.52 mm from the IP. The mean difference between both approaches was significant (*p* < 0.001). A relevant difference between left and right shoulder was not measurable. A Bland–Altman plot was generated for graphical evaluation, which showed comparable results; however, the ascending course of the regression line showed that there were big differences between both methods (Figure 6).

In summary, there was a significantly larger deviation from the IP when using the palpatory approach compared to the ultrasound-guided method. Furthermore, no significant correlation between experience and accuracy could be found. In addition, *t*-test calculations showed that inexperienced users of the ultrasound-assisted method were significantly (*p* < 0.001) closer to the IP than experienced users conducting the conventional palpatory method to determine the IP (Figure 4).

## 4. Discussion

The findings from this prospective study demonstrate that the ultrasound-guided approach for identifying the optimal dorsal puncture point in the glenohumeral joint gap is a secure method. It is especially beneficial for less experienced physicians, who perform punctures more accurately using this method than with the palpatory technique based on anatomical landmarks. A noteworthy and statistically significant difference was observed between the outcomes of both approaches.

Arthrocentesis of the shoulder joint serves as an established diagnostic and therapeutic intervention within the domains of orthopedics, trauma surgery, rheumatology, and radiology. Despite previous investigations demonstrating an accuracy range of 80–94% and establishing the anterior approach as a safe and effective method, the posterior approach method remains the conventional choice in day-to-day practice for both arthrocentesis procedures and arthroscopy. This study used the standard posterior access position to align with common clinical practices [26,27,28]; especially for diagnosis and evaluation of joint empyema, puncture is indispensable [29]. The intra-articular injection of corticosteroids constitutes a scientifically substantiated therapeutic option for addressing adhesive capsulitis, commonly known as “frozen shoulder.” This method consistently demonstrates positive clinical outcomes and is routinely performed as a standard procedure [30]. The intra-articular injection of corticosteroids or hyaluronic acid stands as a conservative treatment option for symptomatic glenohumeral osteoarthritis. It continues to be an established standard procedure with proven efficacy in managing the condition [31]. In 2012, Patel et al. demonstrated on cadavers that the ultrasound-guided technique for estimating the glenohumeral joint is significantly more precise than the palpatory method [32]. In contrast to previously mentioned studies, where only two probands were included, our study was conducted with 54 participants with both little job experience and low expertise regarding puncture of the shoulder. In a prospective randomized study by Gibbons et al. on medium-sized joints (elbow, wrist, or ankle), it was shown that inexperienced physicians (mean sonographically assisted puncture experience: 2.8, or 2.71 for punctures using landmarks) achieved significantly better results with the ultrasound-guided puncture technique than with puncture orientation using anatomical landmarks, which aligns with our study results.

Despite the inhomogeneous distribution of experience between the participants, an average experience of 16.48 punctures of the glenohumeral joint makes them inexperienced investigators, even if there was a relevant discrepancy between both techniques in favor of the ultrasound-guided approach.

This shows that the ultrasound-guided technique is a safe tool for clinical practice, especially for young physicians. Furthermore, there was no significant correlation between the experience and mean deviation. Despite there being a significant difference between palpation and ultrasound, 42% correct positions estimated by ultrasound is an insufficient result, even when correct positions were twice as high compared to the palpatory method. Also, with an average deviation of 17.1 mm from the IP by palpation, it is quite likely that arthrocentesis could not have been performed without relevant risk for periarticular structures or a failed joint injection. Patel et al. showed a clear learning curve during ultrasound-guided examination, while they could not confirm this for the palpatory approach [32].

In general, the efficacy of ultrasound-guided techniques could be increased, for example, by additional training for healthcare practitioners in ultrasound interpretation, and guidance could significantly enhance accuracy. Providing clinicians with comprehensive training programs that include both theoretical knowledge and practical hands-on experience in ultrasound imaging can improve their proficiency in identifying anatomical structures and landmarks accurately. This could involve simulated training scenarios where practitioners can practice ultrasound-guided procedures on realistic phantoms or simulation models under the guidance of experienced instructors.

Furthermore, ongoing professional development and refresher courses could help clinicians stay abreast of advancements in ultrasound technology and refine their skills over time. Continuous education programs could focus on refining scanning techniques, optimizing image acquisition settings, and interpreting ultrasound findings accurately.

Additionally, technological advancements in ultrasound imaging systems may contribute to improved accuracy and precision in position estimation. Investing in high-resolution ultrasound equipment with advanced imaging capabilities, such as real-time 3D imaging, could enhance visualization of anatomical structures and facilitate more accurate localization of target positions. Moreover, incorporating features such as automated image segmentation or augmented reality overlays could assist clinicians in precisely identifying and targeting specific anatomical landmarks during ultrasound-guided procedures.

Collaboration between multidisciplinary teams, including radiologists, sonographers, and clinicians, could also foster knowledge exchange and best practices in ultrasound-guided techniques. Establishing interdisciplinary training programs and regular case review sessions can facilitate shared learning experiences and promote a standardized approach to ultrasound-guided procedures across different healthcare specialties.

Furthermore, conducting further research to validate and refine existing protocols for ultrasound-guided techniques may help identify areas for improvement and optimize clinical practice guidelines. Large-scale prospective studies comparing different ultrasound-guided approaches and assessing their accuracy and clinical outcomes could provide valuable insights into the most effective strategies for positioning estimation.

The ultrasound-guided technique reduced the number of failed punctures, especially by inexperienced physicians [32,33]. Advantages of the ultrasound-guided method compared with CT scan or X-ray diagnostics include radiation-free images [34]. With regard to patient safety, utilizing ultrasound, which does not involve ionizing radiation like CT scans or X-rays do, spares patients from potential harmful effects associated with radiation exposure. This is particularly important for pregnant women, children, and individuals who require frequent imaging studies. Eliminating radiation exposure decreases the risk of developing radiation-induced complications such as cancer. This is especially beneficial for patients undergoing repetitive or long-term monitoring where cumulative radiation exposure can become a concern. Furthermore, ultrasound provides real-time imaging capabilities, allowing physicians to visualize anatomical structures dynamically during procedures. This facilitates accurate needle placement, biopsies, and other interventions, potentially reducing the need for repeated attempts and minimizing procedural time [35]. Another advantage of ultrasound imaging is that ultrasound machines are typically more portable and readily available compared to CT scanners or X-ray machines. This accessibility enables point-of-care imaging, facilitating timely diagnosis and intervention, particularly in emergency or critical care settings. With no requirement for expensive radiation shielding or specialized facilities, ultrasound-guided procedures may be more cost effective compared to CT- or X-ray-guided interventions. This can lead to reduced healthcare costs and improved resource utilization. In summary, ultrasound examinations and interventions can be performed without significant effort or risks for either the examiner or the patient [19,20,24,36,37,38].

Although we did not separately record the time for determining the IP for both the palpatory and the sonographically assisted methods in our study, previous investigations demonstrated that ultrasound-guided aspirations are not only timesaving but also easy to learn [35]. A study in emergency medicine examining the hip, ankle, and wrist joints found that the average time from needle insertion to successful aspiration was shorter in the ultrasound group compared to the group relying on anatomical landmarks [17,39]. Furthermore, studies indicate that ultrasound-guided arthrocentesis by emergency room physicians has a short learning curve. With an average training duration of only 30 to 60 min, study participants achieved nearly perfect success rates [17,39]. In summary, it can be stated that the literature of recent years aligns with the results of the present study regarding efficiency, time savings, and safety.

A limitation of this study is that no actual joint puncture was performed; instead, the focus was on estimating the optimal point of entry. However, it must be emphasized that the starting point for a successful puncture is the entry point. If this location is incorrectly determined, it cannot be assumed that the puncture will be successful. Additionally, the reference point determination was conducted by a single examiner, albeit an experienced one, in both shoulder arthroscopy and ultrasound diagnostics. While the reference point was validated through sonography, it lacked validation through other methods. Furthermore, participants were only exposed to a video illustrating the two approaches, and a live demonstration on a patient would likely have a more profound impact.

Another limitation is the potential constraint arising from the geographical scope of participant recruitment in their study. By restricting inclusion criteria to individuals approbated in Germany, Austria, or Switzerland, the findings may not fully capture the experiences and perspectives of populations outside of these regions. This could raise concerns about the generalizability of the study results to broader populations or different training contexts. Future research could aim to address these limitations by including participants from diverse geographic regions with different training contexts or conducting comparative studies across different locations. By doing so, researchers can enhance the robustness and applicability of their findings, ultimately contributing to a more comprehensive understanding of the topic at hand.

However, it can be assumed that successful joint puncture is attainable following accurate estimation of the entry point. Notably, only the dorsal point of entry was investigated, despite safer and more precise alternative points for puncture existing.

To address these limitations, future studies should explore other puncture techniques using the ultrasound-guided method.

## 5. Conclusions

In conclusion, this study underscores the efficacy of the ultrasound-guided method over the palpatory technique for inexperienced physicians engaged in shoulder joint puncture and arthroscopy. Despite the majority of participants being previously unfamiliar with these procedures, our findings demonstrate that ultrasound guidance not only enhances accuracy in determining the optimal point of entry but also contributes to increased patient and examiner safety by eliminating the need for ionizing radiation. While the ultrasound-guided approach has been recommended by German guidelines and integrated into specialist training since 2015, our study reveals a notable gap in practical application among participants. Therefore, based on the presented results, we advocate for a paradigm shift in clinical practice and highly recommend that inexperienced physicians prioritize ultrasound-guided puncture for shoulder joint procedures.

## Data Availability

The data presented in this study are available on request from the corresponding author. The data are not publicly available due to privacy.
